# Liquid Biopsy-Based Biomolecular Alterations for the Diagnosis of Triple-Negative Breast Cancer in Adults: A Scoping Review

**DOI:** 10.3390/diagnostics16020360

**Published:** 2026-01-22

**Authors:** Orieta Navarrete-Fernández, Eddy Mora, Josue Rivadeneira, Víctor Herrera, Ángela L. Riffo-Campos

**Affiliations:** 1 Universidad de La Frontera, Programa de Doctorado en Ciencias Médicas, Temuco 4780000, Chile; 2 Universidad de Santiago de Chile, Postgrado de Anatomía Patológica, Santiago 8370003, Chile; 3 Instituto de Investigaciones Médicas y Biotecnológicas, Universidad de Carabobo (IIMBUC), Valencia 2001, Venezuela; 4 Hospital Clínico Félix Bulnes, Unidad de Patología Mamaria, Santiago 9110056, Chile; 5 Universidad de La Frontera, Department of Chemical Engineering, Temuco 4780000, Chile; 6 Center for Cancer Prevention and Control (CECAN), Santiago 8331150, Chile

**Keywords:** triple-negative breast cancer, liquid biopsy, circulating biomarkers, protein biomarkers, RNA biomarkers, DNA methylation, microRNAs, diagnostic accuracy, non-invasive diagnostics, molecular oncology

## Abstract

**Background/Objectives:** Triple-negative breast cancer (TNBC) is an aggressive subtype, with limited diagnostic options and no targeted early detection tools. Liquid biopsy represents a minimally invasive approach for detecting tumor-derived molecular alterations in body fluids. This scoping review aimed to comprehensively synthesize all liquid biopsy-derived molecular biomarkers evaluated for the diagnosis of TNBC in adults. **Methods:** This review followed the Arksey and O’Malley framework and PRISMA-ScR guidelines. Systematic searches of PubMed, Scopus, Embase, and Web of Science identified primary human studies evaluating circulating molecular biomarkers for TNBC diagnosis. Non-TNBC, non-human, hereditary, treatment-response, and nonmolecular studies were excluded. Data on study design, patient characteristics, biospecimen type, analytical platforms, biomarker class, and diagnostic performance were extracted and synthesized descriptively by biomolecule class. **Results:** Thirty-two studies met the inclusion criteria, comprising 15 protein-based, 12 RNA-based, and 6 DNA-based studies (one reporting both protein and RNA). In total, 1532 TNBC cases and 3137 participants in the comparator group were analyzed. Protein biomarkers were the most frequently studied, although only APOA4 appeared in more than one study, with conflicting results. RNA-based biomarkers identified promising candidates, particularly miR-21, but validation cohorts were scarce. DNA methylation markers showed promising diagnostic accuracy yet lacked replication. Most studies were small retrospective case–control designs with heterogeneous comparators and inconsistent diagnostic reporting. **Conclusions:** Evidence for liquid biopsy-derived biomarkers in TNBC remains limited, heterogeneous, and insufficiently validated. No biomarker currently shows reproducibility suitable for clinical implementation. Robust, prospective, and standardized studies are needed to advance liquid biopsy-based diagnostics in TNBC.

## 1. Introduction

Breast cancer was the second most frequently diagnosed malignancy worldwide in 2022, second only to lung cancer, with an estimated 2.3 million new cases, representing 11.6% of all new cancer cases. It is the leading cause of cancer-related death in women, accounting for 15.4% of deaths (666,000 deaths) [1]. It is a heterogeneous disease with variable morphological and biological characteristics, resulting in different clinical behaviors and responses to therapy [2]. Immunophenotyping using immunohistochemistry allows for molecular classification based on the expression of nuclear estrogen receptors (ERs), progesterone receptors (PRs), human epidermal growth factor 2 (HER2), and Ki-67, which act as prognostic factors and have predictive values for therapy response. These markers allow breast cancer to be classified into the following subtypes: luminal A (ER and/or PR+, HER2−, Ki-67 low), luminal B (ER and/or PR+, HER2−, Ki-67 high) or (ER and/or PR+, HER2+), HER2-enriched (ER and PR−, HER2+), and triple-negative (ER−, PR−, HER2−). The triple-negative breast cancer (TNBC) subtype does not express any of the aforementioned markers and accounts for 15–20% of breast cancers. These tumors present a wide spectrum of morphologies, with most being high-grade and proliferation indices exceeding 80% [2,3]. They have a more aggressive clinical course, with an earlier age of presentation, a higher potential risk of metastasis, a worse clinical outcome with more frequent relapses, and a lower survival rate. This subtype includes various entities with different genetic, transcriptional, histological, and clinical profiles [4]. Therefore, early diagnosis is fundamental to enable effective and timely treatment with curative aims. Currently, breast cancer diagnosis relies primarily on imaging studies, mainly mammography and ultrasound, the latter being preferentially used in women under 40 years of age. However, mammography has reduced diagnostic performance in younger women due to high breast density, which lowers sensitivity and specificity and may lead to delayed detection and a larger tumor size at diagnosis [5]. Moreover, most screening programs begin at 50 years of age, leaving younger women outside routine screening despite representing a clinically relevant population for early detection [6].

In cases of suspicious findings, an image-guided biopsy is performed to obtain neoplastic tissue that allows for diagnosis and classification. Nonetheless, core biopsy may be limited by tissue volume and sampling representativeness, and diagnostic assessment can be further complicated by tissue fragmentation or distortion. Moreover, histological grade may be underestimated, and intratumoral heterogeneity may be missed, contributing to discordant prognostic or predictive biomarker results in some cases [7,8]. Another point to consider is the presence of access barriers that can delay breast cancer screening and diagnosis. In Latin America, travel distance and mobility constraints may contribute to delays in timely access to diagnostic and therapeutic services [9]. In addition, evidence from a longitudinal primary-care–linked breast cancer cohort shows that the health-system interval can be the longest component of the diagnostic trajectory, largely driven by limited access to diagnostic tests and waiting times [10]. Importantly, inequities may persist even in systems with universal health guarantees. For example, in Chile, administrative data show higher case-fatality ratios and lower survival among publicly insured patients compared with privately insured patients, and better survival for patients living in the Metropolitan Region, which may be partly related to the concentration of specialized centers and specialists in metropolitan areas [11]. Consistently, a real-world Chilean TNBC cohort in the Metropolitan Region reported socioeconomic gradients in presentation and outcomes, including lower screen detection in low-income groups and worse survival patterns aligned with access disparities [12]. 

Therefore, the development of diagnostic techniques that facilitate access and allow for early diagnosis is necessary. Liquid biopsy is a potential diagnostic technique, involving the collection of a sample of bodily fluid (blood, urine, or saliva, for example) and has the potential to allow the evaluation of the presence of tumor derivatives such as DNA, RNA, circulating tumor cells (CTCs), proteins, or extracellular vesicles (EVs) [13]. However, important limitations remain, including the need for standardized pre-analytical and analytical procedures, technically demanding workflows, and biological constraints such as low tumor fraction and high background cfDNA. As a result, liquid biopsy is currently viewed as complementary rather than fully substitutive to tissue-based diagnostics [14]. In early breast cancer, ctDNA often represents < 0.1% of total cfDNA and may be undetectable in approximately 90% of patients receiving neoadjuvant therapy; nonetheless, when detectable, it can precede clinical relapse by a median of ~7.9 months (up to 10.7 months), although detection rates in localized disease may range from ~50% to 62.5% [15].

Taken together, these diagnostic- and access-related challenges, together with the methodological and biological limitations inherent to liquid biopsy approaches, highlight the need for a comprehensive mapping and synthesis of the available evidence on liquid biopsy-derived biomarkers for the diagnosis of TNBC. Although liquid biopsy has been widely studied in breast cancer [16,17], TNBC-specific evidence is limited and largely restricted to reviews focused on selected biomarker types [18,19,20]. In this scoping review, our objective is to synthesize the available evidence on all types of liquid biopsy-derived biomarkers reported for the diagnosis of TNBC up to the end of 2024.

## 2. Materials and Methods

### 2.1. Protocol and Reporting

This scoping review was conducted in accordance with the methodological framework of Arksey and O’Malley and reported following the PRISMA-ScR checklist [21]. The protocol and eligibility criteria were pre-specified prior to study initiation.

### 2.2. Information Sources and Search

Five reviewers (O.N.-F., J.R., E.M., V.H., and A.L.R.-C.) independently developed and tested the search strategy and subsequently reached consensus on the final search strategy, which was then applied in Medline (via PubMed), Scopus, Embase, and Web of Science (WoS) up to 9 October 2024. The search strategies combined controlled vocabulary (MeSH, Emtree, and DeCS) with free-text terms related to “triple-negative breast cancer,” “liquid biopsy,” body fluids (“plasma,” “serum,” “blood,” “saliva,” “extracellular vesicles”), and molecular analytes (DNA, cfDNA, ctDNA, methylation, miRNA, lncRNA, mRNA, proteins/proteomics, lipids/lipidomics, and glycosylation), connected using Boolean operators (AND/OR/NOT). Reference lists of all included studies were also manually screened to identify additional relevant articles. Full search strategies are provided in Supplementary Table S1.

### 2.3. Eligibility Criteria

We included primary human studies in adults (≥18 years) that enrolled patients with triple-negative breast cancer (TNBC) and evaluated molecular alterations detectable in liquid biopsy (circulating-tumor-derived biological material in body fluids) exclusively in relation to diagnosis, without language restrictions. Excluded: in vivo/in vitro/in silico-only work or methodological study, without a validation cohort; case reports, tissue-only analyses, no liquid biopsy (not reflecting tumor-derived liquid biopsy signals), not molecular biomarkers, or not diagnostic molecular biomarkers; reviews, editorials, and letters; studies without a TNBC subgroup or in which data for TNBC could not be identified; analyses restricted to circulating tumor cell (CTC) counts without molecular characterization; hereditary or constitutional/germline studies; and studies focused on metastatic disease, treatment response, or non-diagnostic TNBC focus.

### 2.4. Study Selection and Data Extraction

Three reviewers (O.N.-F., J.R., and E.M.) independently screened titles and abstracts, with disagreements resolved by a third reviewer (A.L.R.-C.), with expertise in cancer molecular biology. Full-text assessment and data extraction were conducted by two reviewers (O.N.-F., and E.M.), both board-certified anatomical pathologists with expertise in breast pathology. Extracted data items included DOI, authorship, year, country, study design, sample size and age, biospecimen type, analytical technique, biomarker type, biomarker name/gene symbol, alteration type (e.g., hypermethylation, up-/down-regulation), and diagnostic performance metrics (*p*-values, AUC, sensitivity, specificity when reported). Extracted datasets are presented in Supplementary File S1 (Sheets A–E).

### 2.5. Synthesis of Results

Given heterogeneity across biomarkers, analytical platforms, comparators, and endpoints, results were synthesized descriptively and grouped by analyte or biomolecule class: DNA-, RNA-, and Protein-based. Emphasis was placed on studies reporting diagnostic performance (AUC, sensitivity, specificity). Where available, pre-analytical and analytical factors were also noted.

## 3. Results

### 3.1. Study Selection and Characteristics

**Figure 1:**
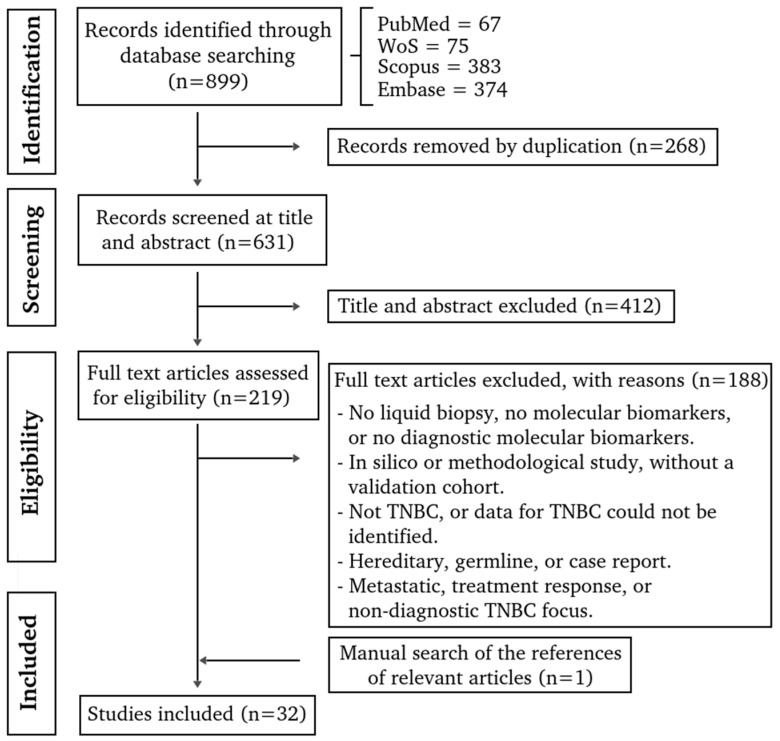
PRISMA flow diagram of the scoping review process.

A total of 899 publications up to 9 October 2024 were identified (Figure 1). Duplicated articles (*n* = 268) and those that did not meet the inclusion criteria during the initial screening of titles and abstracts (*n* = 412), and full-text analysis (*n* = 188) were excluded (Supplementary File S1, Sheets A–C). Additionally, one article was incorporated by hand searching, by checking the reference lists of relevant studies (Supplementary File S1, Sheet D). Finally, 32 studies were examined in this scoping review (Supplementary File S1, Sheet E).

The study design was defined in 28 studies, with 19 case–control (including variants such as nested or retrospective case–control), and the remaining were described as pilot (*n* = 3, including variants), experimental (*n* = 2), cohorts (*n* = 2), cross-sectional, and multi-phase. The studies were conducted primarily in Asia (*n* = 17), followed by Europe (*n* = 7), North America (*n* = 4), Africa (*n* = 2), and South America (*n* = 2). Among the 32 eligible studies, serum was the most commonly analyzed biological specimen (*n* = 17), followed by plasma (*n* = 11), whole or peripheral blood fractions (*n* = 3), buffy coat (*n* = 1), and a few mixed sample types. Finally, protein-based biomarkers were the most frequently investigated, reported in 15 studies, followed by RNA-based biomarkers in 12 studies, whereas DNA-based molecular biomarkers were evaluated in only 6 studies [Table 1]. In one study, both protein-based and RNA-based biomarkers were reported. Of the 32 included studies, 17 reported the area under the receiver operating characteristic curve (AUC), while 14 and 13 studies provided data on sensitivity and specificity, respectively. Although protein-based studies were the most frequently reported, they largely identified single-study biomarkers with limited replication. RNA-based studies, particularly those focusing on miRNAs, showed comparatively greater consistency across independent studies, whereas DNA-based studies were fewer and mainly focused on methylation-based alterations, occasionally reporting higher diagnostic performance metrics but with limited external validation.

### 3.2. Patients’ Characteristics

Across the 32 studies, a total of 1532 TNBC cases and 3137 participants in the comparator group were analyzed. Comparator groups included healthy controls, benign breast disease, non-TNBC breast cancer, non-breast cancer participants, and, in one study, disease-free individuals. The approximate modal age was 52 years, with the majority of studies reporting mean or median ages within the 50–55-year range, with ages ranging from 21 to 93 years. Across the 32 studies, the median sample sizes were 29 TNBC cases and 48.5 controls per study. Regarding comparators, the most common study setups contrasted TNBC vs. healthy controls and TNBC vs. other breast cancer subtypes (e.g., luminal, HER2-positive); fewer studies included benign breast lesions or mixed comparator groups.

### 3.3. Novel Protein-Based Molecular Biomarkers Associated with TNBC Diagnosis

The fifteen studies reporting novel protein-based molecular biomarkers associated with TNBC diagnosis (including one mixed Protein + RNA study, Table 1) were published between 2012 and 2024. The studies included 621 patients with TNBC and 1664 compared group, for an overall cohort of 2285 participants. Across the studies with available data, the reported age range spanned from 26 to 86 years, the mean and modal ages were approximately 52 years. Only one study provided separate age distributions for TNBC and control groups, reporting a mean age of 43.7 ± 7.8 years for TNBC patients and 46.1 ± 10.4 years for controls (Table 1, Study [25]).

Most studies analyzed serum samples (*n* = 12), followed by plasma (*n* = 2), and whole blood fractions (*n* = 1). The predominant analytical techniques included ELISA-based assays, antibody microarrays, and proteomic mass spectrometry approaches (e.g., 2D-DIGE, MALDI-TOF-MS, LC–MS/MS, SWATH). These methodologies primarily aimed to identify circulating proteins differentially expressed in TNBC patients relative to healthy individuals or non-TNBC subtypes.

Among the 15 protein-based studies, at least 69 individual protein biomarkers were identified (Table 1), which could be grouped into 13 functional families, including apolipoproteins, complement components and regulators, immunoglobulins/autoantibodies, coagulation and protease inhibitors, acute-phase/transport proteins, extracellular matrix and adhesion molecules, growth factors/cytokines, and various signaling, enzymatic, and transcriptional regulators. Only APOA4 was reported in three independent studies, while TTR, FN1, APOC1, C3, and C9, were reported in two studies each. All other proteins were described in single studies.

### 3.4. Novel RNA-Based Molecular Biomarkers Associated with TNBC Diagnosis

Twelve studies investigated RNA-based molecular biomarkers (including one mixed Protein + RNA study). Across these investigations, the cumulative sample comprised 550 TNBC cases and 1009 participants in the comparator group, with mean per-study sample sizes of ~48 TNBC cases and ~84 comparator participants. Publication years ranged from 2015 to 2023 (mean 2018). Diagnostic performance metrics were variably reported: AUC was provided in nine studies, while sensitivity and specificity were each reported in six studies. Serum and plasma were the most frequently analyzed biological specimens, whereas urine was used in only one study, and RT-qPCR/qRT-PCR approaches predominated, often combined with discovery arrays. Among the 12 RNA-based studies, five reported multiple RNAs or RNA panels, while seven focused on individual RNA biomarkers, resulting in a total of 40 unique RNAs identified as differentially expressed biomarkers in TNBC (Table 1). Among candidate RNAs, miR-21 was the most frequently investigated across studies (*n* = 4), followed by miR-155 (*n* = 2), whereas other microRNAs (e.g., miR-126-5p, miR-205, miR-199a-5p) and lncRNAs (e.g., ANRIL, SOX2OT, ANRASSF1, UCA1, HIF1A-AS2, NRIL) were each reported by single studies.

### 3.5. Novel DNA-Based Molecular Biomarkers Associated with TNBC Diagnosis

A total of six studies investigated DNA-based molecular biomarkers associated with TNBC diagnosis, encompassing 389 patients with TNBC and 432 participants in the comparator group. Publication years ranged from 2015 to 2023 (mean 2020). Most studies were based on plasma-derived DNA samples, with volumes ranging from 5 to 20 mL, although buffy coat and serum extracellular vesicle (EV) DNA were also analyzed. The most frequently used analytical approaches included Illumina 450K/EPIC methylation arrays, methylation-specific PCR (MSP), digital droplet PCR (ddPCR), and next-generation sequencing (NGS) platforms.

Collectively, 26 unique genes were reported across studies (Table 1). Methylation-based biomarkers, reported in four studies, included cfDNA differentially methylated regions in *SPAG6*, *IFFO1*, *SPHK2*, *TBCD*/*ZNF750*, *LINC10606* and *CPXM1*, as well as promoter methylation of *APC*, *RARB2*, *LINC00299*, and *ADAM12*. Mitochondrial DNA variants were identified in *MT-ND1*, *MT-ND2*, *MT-ND3*, *MT-ND4*, *MT-ND4L*, *MT-ND5*, *MT-ND6*, *MT-CYTB*, *MT-CO1*, *MT-CO2*, *MT-CO3*, *RNR2*, *MT-ATP6*, and *MT-ATP8* (one study); and somatic mutations were examined in *PIK3CA* (one study). Only one study reported AUC (0.74) along with sensitivity (86%) and specificity (90%).

## 4. Discussion

This scoping review synthesizes, for the first time to our knowledge, all molecular classes of liquid biopsy-derived biomarkers evaluated specifically for the diagnosis of TNBC. Across 32 primary studies published between 2012 and 2024, we identified proteins, RNAs, and DNA alterations as the three major biomarker categories investigated. Collectively, these findings underscore both the growing interest in minimally invasive diagnostics for TNBC and the considerable methodological and biological gaps that continue to limit their clinical translation.

A consistent observation across studies was the substantial heterogeneity in biomarker biology, study design, and analytical methods. This variability reflects not only the intrinsic molecular complexity of TNBC [54] but also differences in sample processing, control groups, platforms, and reporting practices [55]. Protein-based biomarkers constituted the most frequently explored category (*n* = 15 studies), followed by RNA-based biomarkers (*n* = 11), while DNA-based biomarkers were less commonly evaluated (*n* = 6). Among RNA-derived candidates, miRNAs demonstrated comparatively stronger replication across independent studies. In contrast, somatic mutations were the least represented biomarker class (*n* = 1).

Despite being the predominant biomarker category, protein-based studies showed limited reproducibility. Only APOA4, whose precise biological function remains incompletely understood, was replicated in more than one independent study [25,26,34]; however, the direction of change was inconsistent. In two studies, APOA4 was significantly up regulated (*p* < 0.05) [25,26], whereas Santana et al. (2024) reported significant down regulation (*p* < 0.05) [34]. These discrepancies highlight the sensitivity of protein measurements to pre-analytical workflows, comparator group selection, and population-level variability, reinforcing the need for standardized approaches to quantify spatial, temporal, and inter-individual heterogeneity [55,56].

RNA-based biomarkers included several promising candidates, most notably miR-21, which was significantly up regulated in three of the four studies analyzed [38,40,43,47]. Biologically, the recurrent detection of miR-21 in TNBC is plausible. miR-21 functions as an oncomiR by directly targeting PTEN, thereby promoting activation of the PI3K/AKT signaling pathway, and has been associated with STAT3- and TGF-β–driven pro-invasive and pro-tumorigenic programs [57]. However, it has been validated in a limited number of samples, raising concerns about its reproducibility, generalizability, and suitability as a clinically reliable diagnostic biomarker [56]. Additional RNAs, including miR-199a-5p, miR-155, and miR-205 [38,40], as well as lncRNAs such as NRIL, HIF1A-AS2, and UCA1 [41], also demonstrated excellent diagnostic performance. Notably, miR-199a-5p achieved an individual AUC of 0.8838 [38], whereas the remaining markers, evaluated as part of multi-RNA panels, yielded AUC values exceeding 0.96 [40]. However, these values were reported in single studies only, underscoring the lack of external validation and the early-stage nature of this research field.

Among DNA-based biomarkers, only one study reported an AUC for a methylation panel [48] including *SPAG6*, *LINC10606*, and *TBCD/ZNF750*, which showed limited diagnostic performance (AUC: 0.74 in the validation set). The other three studies that analyzed methylation did not report AUC values. The 26 reported genes or loci primarily represent methylation-based classification signatures selected to distinguish TNBC from heterogeneous comparator groups, rather than recurrent TNBC-specific driver events. Although several loci map to pathways broadly involved in breast cancer biology, TNBC specificity remains largely unestablished. Nonetheless, some markers show biological plausibility in aggressive disease contexts, such as *SPHK2*, linked to pro-metastatic signaling [58], and *ZNF750*, associated with invasion-suppressive programs [59]. The absence of *PIK3CA* hotspot mutations in plasma is biologically consistent with their lower prevalence in TNBC compared with luminal subtypes [60]. To date, none were replicated across independent populations, batches, or analytical platforms.

When considered comparatively, protein-, RNA-, and DNA-based biomarkers capture complementary layers of TNBC biology but differ markedly in evidentiary maturity. Protein-based biomarkers dominate the literature, reflecting technical accessibility and established serum assays, yet they show the poorest reproducibility across studies. RNA-based biomarkers, particularly circulating miRNAs, exhibit comparatively greater consistency across independent cohorts, suggesting higher analytical robustness despite fewer studies. DNA-based biomarkers, mainly methylation-based alterations, occasionally report higher diagnostic performance metrics, but remain sparsely studied and largely unreplicated. The predominance of single-study biomarkers across all molecular classes reflects a fragmented evidence base where most discoveries remain unvalidated and therefore unsuitable for immediate clinical translation.

Beyond biomarker biology, study design limitations were pervasive. Most investigations employed retrospective case–control designs, which were vulnerable to spectrum bias and frequently overestimated diagnostic performance [61,62]. Diagnostic performance was inconsistently reported, with only 17 studies providing AUC values and even fewer reporting sensitivity or specificity. Validation cohorts were uncommon, and independent testing sets were rarely implemented. Clinical comparators were heterogeneous, often combining healthy individuals with patients with non-TNBC breast cancer, and demographic matching was generally inadequate, with only one study reporting age distributions separately for cases and controls. Sample sizes were typically small (median 29 TNBC cases and 35 controls), restricting subgroup analyses and limiting statistical robustness. Collectively, these issues hinder reproducibility, generalizability, and the translational potential of the biomarkers identified. Accordingly, the current body of evidence should be regarded as exploratory and hypothesis-generating rather than clinically definitive.

### Strengths and Limitations

Strengths of this scoping review include a comprehensive multi-database search strategy, adherence to PRISMA-ScR guidelines, independent screening and data extraction, and structured reporting by biomarker class. However, limitations inherent to scoping reviews also apply: no meta-analysis was conducted, and the high heterogeneity across studies prevented quantitative synthesis of diagnostic accuracy. Because only published studies were included, publication bias cannot be ruled out.

From a clinical perspective, the primary limitation is the lack of validation in independent or prospective cohorts. Most biomarkers were evaluated exclusively in discovery populations, making their real-world diagnostic utility uncertain. Until robust external replication is achieved, none of the identified circulating biomarkers (protein-, RNA-, or DNA-based), can be considered ready for clinical implementation in TNBC diagnosis. Overall, these findings indicate that current translational barriers are driven primarily by methodological fragmentation, rather than by intrinsic limitations of any single biomarker class.

From a biological perspective, circulating biomarkers detected by liquid biopsy may originate not only from tumor cells but also from non-malignant components of the tumor microenvironment, including immune, stromal, and endothelial cells. In TNBC, which is characterized by prominent immune infiltration and stromal interactions, inflammatory and immune-related processes can substantially influence circulating biomolecule levels. As a result, some reported biomarkers may reflect tumor–host interactions rather than tumor-specific alterations, potentially reducing diagnostic specificity, particularly in early-stage disease or in the absence of tumor-enriched validation. Because most included studies did not specifically assess cellular origin, the relative contribution of tumor- versus microenvironment-derived signals could not be systematically evaluated within the scope of this review.

## 5. Conclusions

This scoping review synthesizes the existing evidence on liquid biopsy-derived molecular biomarkers for the diagnosis of triple-negative breast cancer up to 2024. Although numerous protein-, RNA-, and DNA-based candidates have been proposed, the current evidence remains constrained by methodological heterogeneity, small cohorts, inconsistent reporting, and a lack of external and prospective validation. At present, no biomarker demonstrates sufficient reproducibility or robustness for clinical diagnostic implementation in TNBC. Advancing the field will require well-designed, adequately powered, and standardized multi-phase studies that incorporate independent validation cohorts and harmonized pre-analytical and analytical protocols.

## Figures and Tables

**Table 1:** Molecular biomarkers associated with diagnostic of TNBC.

Biomarker Category	Biomarker(s)	Alteration Type	Sample Size	*n* Cases	*n* Controls	Biofluid	Detection Method	AUC	*p*-Value	Ref.
Protein	Panel: KIT, ITGB1, EFNA5, SRP54, FAS, BRCA1, XBP1, and others	Up-regulated	56	28	28	Plasma	Antibody microarray	Various	*p* < 0.05	[22]
Protein	TTR, SERPINA1, HP	Up-regulated; Down-regulated	60	30	30	Serum	2D-DIGE; MALDI-TOF-MS	NR	*p* < 0.05	[23]
Protein	FN1, A2M, C4BPA	Up-regulated	28	8	20	Plasma	iTRAQ; WB; ELISA	0.853	A2M, C4BPA: *p* < 0.0001; FN1: *p* < 0.018	[24]
Protein	Panel: CPN2, CO2, MYL6, HV101, APOA4, PI16, CXCL7, VTDB, IGJ, KNG1	Up-regulated; Down-regulated	39	19	20	Serum	2D-DIGE/MALDI; iTRAQ-LC-MS/MS; SWATH; WB; SRM	NR	*p* < 0.05	[25]
Protein	Panel: APO1, CFH, VTNC, C3, C4A, C9, LGALS3BP, FCN3, RBP4, FN1, APOA4, ORM1, ZPI, TTR, APOC1, APOC3, IGHM, IG chains	Up-regulated; Down-regulated	16	8	8	Serum	NP exposure; SDS-PAGE; LC-MS/MS; SWATH/SRM	NR	*p* < 0.05	[26]
Protein	MR	Glycosylation pattern change	55	35	110 *	Serum	IP; SDS-PAGE; HILIC; MALDI-TOF-MS	NR	*p* < 0.01	[27]
Protein	VEGF	Increased serum concentration	65	30	35	Serum	ELISA	NR	*p* = 0.01 (size) *p* = 0.03 (stage)	[28]
Protein	RAI14	Increased serum concentration	106	46	60 *	Serum	ELISA	0.934	*p* < 1 × 10^−4^	[29]
Protein	ApoC-I	Increased serum concentration	380	165	215 *	Serum	SELDI-TOF-MS; qRT-PCR; ELISA; WB	0.908	*p* < 1 × 10^−4^	[30]
Protein	KJ901215, FAM49B, HYI, GARS, CRLF3	Lower concentration panel	389	123	776 *	Serum	Serology	0.875	*p* < 0.05	[31]
Protein	ANXA2	Higher circulating concentrations	126	58	179 *	Serum	Western blot; ELISA	1	*p* < 1 × 10^−4^	[32]
Protein	GDF15, PKM, SPARC, CA125, WFDC2, COL1A1, FN1, CTGF, S100A7, SPP1, CCL5, hsa-miR-135b, Anti-TP53, HOXA5, SFRP1	Minimal diagnostic performance	115	28	87	Blood	ELISA	TP53 ≤ 0.63	Non sig.	[33]
Protein	ApoA1, ApoA2, ApoC2, ApoC4, C3, CFB, IGLC2/3, GC, PLG, SERPINA3, IGHC1, C9, LRG1, C4B	Panel changes	123	20	204 *	Plasma	Ultracentrifugation; LC-MS/MS	Various	Various	[34]
Protein	TRAF6	Higher serum concentration	39	13	61 *	Serum	ELISA	NR	*p* = 0.010	[35]
Protein	Anti-TXNL2	Higher concentration	20	10	10	Serum	HuProt microarray	NR	NR	[36]
RNA (lncRNA)	*ZFAS1*	Up and down regulated	80	40	40	Peripheral blood	RT2 lncRNA PCR Array; qRT-PCR	NR	*p* < 1 × 10^−4^	[37]
RNA (miRNA)	miR-199a-5p, miR-16, miR-21	Down regulated	327	72	255 *	Plasma	miRNA arrays; RT-qPCR	0.88	*p* < 0.0001	[38]
RNA (miRNA)	miR-126-5p	Concentration change	42	21	21	Plasma	Microarray; RT-qPCR	0.814	*p* = 1.4 × 10^−5^	[39]
RNA (miRNA)	miR-21, miR-155, miR-205	Up and down regulated	190	139	51	Serum	RT-qPCR	0.961	*p* < 1 × 10^−4^	[40]
RNA (lncRNA)	*NRIL*, *HIF1A-AS2*, *UCA1*	Up regulated	100	25	75 *	Serum	Microarray; RT-qPCR	0.934	*p* < 0.01	[41]
RNA (miRNA)	miR-25-3p	Up regulated	81	12	69 *	Serum	NanoString; nCounter	0.74	*p* ≤ 0.05	[42]
RNA (miRNA)	miR-21	Up regulated	50	4	46 *	Plasma	qRT-PCR	NR	NR	[43]
RNA (miRNA)	Initial and diagnostic panel	Serum differential concentration	127	36	91 *	Plasma	RT-qPCR	Panel = 0.929	*p* = 0.0008–0.02	[44]
RNA (miRNA)	miR-135b	Minimal diagnostic performance	115	28	87	Blood	ELISA; RT-qPCR	NR	Non sig.	[33]
RNA (miRNA)	miR-376c, miR-155, miR-17a, miR-10b	Up regulated	71	37	34	Blood	RT-qPCR	0.785	*p* < 0.0001	[45]
RNA (lncRNA)	*ANRIL*, *SOX2OT*, *ANRASSF1*	Up regulated	340	120	220 *	Plasma	qRT-PCR	0.959	ANRIL: *p* < 0.01; others: *p* < 0.05	[46]
RNA (miRNA)	Serum: let-7a, let-7e, miR-21, miR-15a, miR-17, miR-18a, miR-19b, miR-30b, *GlyCCC2*; Urine: miR-18a, miR-19b, miR-30b, miR-222, miR-320, *GlyCCC2*	Increase and decrease in concentration	36	16	20	Serum; Urine	RT-qPCR	NR	*p* < 0.05	[47]
DNA (cfDNA methylation)	*SPAG6*, *IFFO1*, *SPHK2*; *TBCD*/*ZNF750*, *LINC10606*, *CPXM1*	Hypermethylation and hypomethylation	223	139	84	Plasma	Illumina 450K/EPIC; XGBoost; ddPCR	Test = 0.78; Validation = 0.74	*p* < 0.0001	[48]
DNA (DNA methylation)	Promoter methylation: *APC*, *RARB2*	Promoter methylation (RARB2 methylated in TNBC)	216	71	145 *	Serum	MSP	NR	*p* = 0.007 (RARB2)	[49]
DNA (Mutations)	*PIK3CA* hotspot mutations	No plasma mutations	32	10	22	Plasma	RT-PCR	NR	---	[50]
DNA (Methylation)	*LINC00299* (cg06588802)	Hypermethylation (leukocyte DNA)	313	154	159	Buffy coat	ddPCR	NR	*p* = 0.0025; *p* = 0.001 (tertile 1)	[51]
DNA (mtDNA)	mtDNA variants (*ND1*, *ND2*, *ND3*, *ND4*, *ND4L*, *ND5*, *ND6*, *CYTB*, *CO1*, *CO2*, *CO3*, *RNR2*, *ATP6*, *ATP8*)	EV concentration; tumor-specific/shared EV mutations	18	9	9	Serum	NGS (Illumina NovaSeq, PE150)	NR	*p* < 0.0001 (EV concentration)	[52]
DNA (cfDNA methylation)	Promoter methylation: *ADAM12*	Hypomethylation	19	6	13	Plasma	Illumina 450K; Pyrosequencing	NR	NR	[53]

All cases were TNBC patients; * may include healthy controls, not BC, non-TNBC, benign breast disease or free disease and luminal breast cancer; if not *, all controls were not BC participants; NR; Not reported. TNBC, Triple negative breast cancer; BC, Breast cancer; KIT, Mast/stem cell growth factor receptor; ITGB1, Integrin beta-1; EFNA5, Ephrin-A5; SRP54, Signal recognition particle 54 kDa; FAS, TNFR superfamily member 6 (Ab1); BRCA1, Breast and ovarian cancer susceptibility protein 1; XBP1, X box-binding protein 1; TTR, Transthyretin; SERPINA1, Alpha-1-antitrypsin; HP, Haptoglobin; FN1, Fibronectin; A2M, Alpha-2-macroglobulin; C4BPA, Complement component-4-binding protein-alpha; CFB, Complement factor-B; CPN2, Carboxypeptidase N subunit 2; CO2, Carbon dioxide; MYL6, Myosin light chain 6; HV101, Voltage-gated hydrogen channel 1; APOA4, Apolipoprotein A4; PI16, Peptidase inhibitor 16; CXCL7, Chemokine C-X-C motif Ligand 7; VTDB, Vitamin D-binding protein; IGJ, Immunoglobulin J chain; KNG1, Kininogen-1; APOL1, Apolipoprotein 1; CFH, Complement factor H-related; VTNC, Vitronectin; C3, Complement C3; C4A, Complement C4-A; C9, Complement C9; LGALS3BP, Galectin-3-binding protein, FCN3, Ficolin-3; RBP4, Retinol binding protein 4; ORM1, Orosomucoid; ZPI, protein Z-dependent protease inhibitor; APOC1, apolipoprotein C-I; APOC3, apolipoprotein C-III; IGHM, Immunoglobulin heavy constant mu; IG chains, Immunoglobulin chains; MR, Mannose receptor; MRC1, Mannose receptor C-type 1; VEGF, Vascular endothelial growth factor; CA15-3, Cancer antigen 15.3; RAI14, Retinoic acid induced 14; FAM49B, Family with sequence similarity 49, member B; HYI, Hydroxypyruvate isomerase; GARS, Glycyl-tRNA synthetase 1; CRLF3, Cytokine receptor-like factor 3; ANXA2, Annexin A2; GDF15, Growth differentiation factor 15; PKM, Pyruvate Kinase muscle; SPARC, Osteonectin; CA125, Cancer antigen 125; WFDC2, Human epididymis protein 4; COL1A1, Collagen type 1 alpha 1; CTGF, Connective tissue growth factor; S100A7, Psoriasin; SPP1, Osteoponin; CCL5, RANTES; HOXA5, Homeobox 5; SFRP1, Secreted frizzled-related protein 1; IGLC2/3, Immunoglobulin lambda constant 2/3; GC, Vitamin D-binding protein; PLG, Plasminogen; SERPINA3, Serpin family A member 3; IGHC1, Immunoglobulin Heavy Constant Gamma 1; C9, Complement Component 9; LRG1, Leucine-rich alpha-2-glycoprotein 1; C4B, Complement C4B; TWEAK, (TNF)-like weak inducer of apoptosis; TRAF6, TNF receptor-associated factor 6; Anti-TXNL2, Thioredoxin-like 2 autoantibody; lncRNA, long non-coding RNA; miRNA, microRNA; cfDNA, Circulating cell-free DNA; *SPAG6*: Sperm Associated Antigen 6; *IFFO1*, Intermediate Filament Family Orphan 1; *SPHK2*, Sphingosine Kinase 2; *TBCD*, Tubulin-specific chaperone D; *ZNF750*, Zinc Finger Protein 750; *LINC10606*, Long Intergenic Non-Protein Coding RNA 606; *CPXM1*, Carboxypeptidase X, M14 Family Member 1; *APC*, Adenomatous polyposis coli; *RARB2*, Retinoic Acid Receptor Beta; *PIK3CA*, Phosphatidylinositol-4,5-Bisphosphate 3-Kinase Catalytic Subunit Alpha; *LINC00299*, Long Intergenic Non-Protein Coding RNA 299; *ND1*, Ubiquinone Oxidoreductase Core Subunit 1; *ND2*, Ubiquinone Oxidoreductase Subunit 2; *ND3*, Ubiquinone Oxidoreductase Subunit 3; *ND4*, Ubiquinone Oxidoreductase Subunit 4; *ND4L*, Ubiquinone Oxidoreductase Subunit 4L; *ND5*, Ubiquinone Oxidoreductase Subunit 5; *ND6*, Ubiquinone Oxidoreductase Subunit 6; *CYTB*, *Cytochrome b* (complex III); *CO1*, Cytochrome c Oxidase Subunit 1; *CO2*, Cytochrome c Oxidase Subunit 2; *CO3*, Cytochrome c Oxidase Subunit 3; *RNR2*, 16S Ribosomal RNA; *ATP6*, ATP Synthase F0 Subunit 6; *ATP8*, ATP Synthase F0 Subunit 8; *ADAM12*, ADAM Metallopeptidase Domain 12.

## Data Availability

The original contributions presented in this study are included in the article/Supplementary Material. Further inquiries can be directed to the corresponding author.

## Supplementary Materials

The following supporting information can be downloaded at https://www.mdpi.com/article/10.3390/diagnostics16020360/s1, Table S1: Search strategy 10 October 2024. File S1: Supplementary File detailing the scoping review process, including (A) Identification, (B) Screening, (C) Eligibility, (D) Manual Search, and (E) Data Extraction.

## Abbreviations

The following abbreviations are used in this manuscript:

AUC	Area under the receiver operating characteristic curve
BC	Breast cancer
cfDNA	Cell-free DNA
CTC	Circulating tumor cell
ctDNA	Circulating tumor DNA
ddPCR	Digital droplet polymerase chain reaction
ELISA	Enzyme-linked immunosorbent assay
EVs	Extracellular vesicles
HILIC	Hydrophilic interaction liquid chromatography
lncRNA	Long non-coding RNA
MALDI-TOF-MS	Matrix-assisted laser desorption/ionization time-of-flight mass spectrometry
miRNA	MicroRNA
MSP	Methylation-specific polymerase chain reaction
NGS	Next-generation sequencing
RT-qPCR	Reverse transcription quantitative polymerase chain reaction
SDS-PAGE	Sodium dodecyl sulfate polyacrylamide gel electrophoresis
SRM	Selected reaction monitoring
SWATH	Sequential window acquisition of all theoretical fragment ion spectra
TNBC	Triple-negative breast cancer
WB	Western blot
