# Supplementary material for: Liquid Biopsy-Based Biomolecular Alterations for the Diagnosis of Triple-Negative Breast Cancer in Adults: A Scoping Review
Source: Diagnostics (Basel). 2026 Jan 22;16(2):360. doi: 10.3390/diagnostics16020360 (PMC12840333; doi:10.3390/diagnostics16020360)
Supplement: Supplementary file 1 [file diagnostics-16-00360-s001.zip › Supplementary Table S1.pdf]

Supplementary Table S1. Search strategy October 10, 2024

Resourse	Search strategy	N of paper
PubMed	(("diagnosis"[Title/Abstract] OR "diagnostic"[Title/Abstract]) AND ("triple negative breast cancer"[Title/Abstract] OR "basal like breast cancer"[Title/Abstract]) AND ("gene"[Title/Abstract] OR "target"[Title/Abstract] OR "protein"[Title/Abstract] OR "biomarker"[Title/Abstract] OR "RNA"[Title/Abstract] OR "methylation"[Title/Abstract] OR "molecular profile"[Title/Abstract] OR "DNA"[Title/Abstract] OR "circulating"[Title/Abstract] OR "mutation"[Title/Abstract] OR "exosome"[Title/Abstract]) AND ("liquid biopsy"[All Fields] OR "blood"[All Fields] OR "plasma"[All Fields] OR "urine"[All Fields] OR "saliva"[All Fields])) NOT ("review"[Publication Type] OR "in vitro"[All Fields] OR "in silico"[All Fields] OR "in vivo"[All Fields] OR "animal model"[All Fields] OR "cell line*"[All Fields] OR "xenograft"[All Fields])	67
Scopus	TITLE-ABS-KEY ("diagnosis") OR TITLE-ABS-KEY ("diagnostic") AND TITLE-ABS-KEY ("triple negative breast cancer") OR TITLE-ABS-KEY ("basal like breast cancer") AND TITLE-ABS-KEY (gene) OR TITLE-ABS-KEY (target) OR TITLE-ABS-KEY (protein) OR TITLE-ABS-KEY (rna) OR TITLE-ABS-KEY (biomarker) OR TITLE-ABS-KEY (methylation) OR TITLE-ABS-KEY ("molecular profile") OR TITLE-ABS-KEY (DNA) OR TITLE-ABS-KEY (circulating) OR TITLE-ABS-KEY (mutation) OR TITLE-ABS-KEY (exosome) AND ALL ("liquid biopsy") OR ALL ("blood") OR ALL ("plasma") OR ALL ("urine") OR ALL ("saliva") AND NOT ALL ("in vitro") OR ALL ("in vivo") OR ALL ("in silico") AND NOT ALL ("animal model") OR ALL ("cell line*") OR ALL (xenograft) AND (LIMIT-TO (DOCTYPE,"ar"))	383
Embase	('diagnosis' OR 'diagnostic') AND ('triple negative breast cancer' OR 'basal like breast cancer') AND (gene OR target OR protein OR biomarker OR rna OR methylation OR 'molecular profile' OR 'dna' OR 'circulating' OR 'mutation' OR 'exosome') AND ('liquid biopsy' OR 'blood' OR 'plasma' OR 'urine' OR 'saliva') NOT ('cell line*' OR 'animal model' OR 'in vitro' OR 'xenograft') AND 'article'/it	387
WOS	TS=("diagnosis" OR "diagnostic") AND TS=("triple negative breast cancer" OR "basal like breast cancer") AND TS=(Gene OR RNA OR target OR protein OR methylation OR “molecular profile” OR Biomarker OR DNA OR circulating OR mutation OR exosome) AND TS=("liquid biopsy" OR "blood" OR "plasma" OR "urine" OR "saliva") NOT SO="review" NOT SO="letter" NOT SO="correspondence as topic" NOT SO="comments" NOT SO="opinion" NOT TS=("in vitro" OR "in vivo" OR “animal model” OR xenograft OR “cell line*”)<br>+ aditional filter Document types: Article	75
